# The association between evidence of a predator threat and responsiveness to alarm calls in Western Australian magpies (*Cracticus tibicen dorsalis*)

**DOI:** 10.7717/peerj.7572

**Published:** 2019-08-26

**Authors:** Annabel Silvestri, Kate Morgan, Amanda R. Ridley

**Affiliations:** School of Biological Sciences, University of Western Australia, Perth, WA, Australia

**Keywords:** Alarm call, Social information, Magpies, Vigilance, Predators

## Abstract

Alarm calls are a widespread form of antipredator defence and being alerted to the presence of predators by the alarm calls of conspecifics is considered one of the benefits of group living. However, while social information can allow an individual to gain additional information, it can also at times be inaccurate or irrelevant. Such variation in the accuracy of social information is predicted to select for receivers to discriminate between sources of social information. In this study, we used playback experiments to determine whether Western Australian magpies (*Cracticus tibicen dorsalis*) respond to the predator information associated with alarm calls. Magpies were exposed to the alarm calls of two group members that differed in the threat associated with the alarm call: one call was played in the presence of a predator model while the other was not—in order to establish differences in the predator information provided by each caller. We then played back the alarm calls of the same group members in the absence of the predator model to determine whether magpies responded differently to signallers in response to the *previous* association between the alarm call and a predator threat. We found that receivers showed significantly greater levels of responsiveness to signallers that previously gave alarm calls in the appropriate context. Thus, the accuracy of threat-based information influenced subsequent receiver response.

## Introduction

Cooperation in group-living species provides a number of benefits to group members. These include cooperative territorial defence (Christensen & Radford, 2018), improved detection of threats (Elgar, 1989; Ridley, Raihani & Bell, 2010) and shared vigilance (Treves, 1998; Bell et al., 2009). Shared vigilance benefits group members, as it allows individuals to scan for predators less frequently and dedicate more time to other activities, such as foraging and offspring care (Elgar, 1989; Roberts, 1996; Ridley, Raihani & Bell, 2010; Ridley, Nelson-Flower & Thompson, 2013).

Another proposed benefit of group-living is greater access to social information (Danchin et al., 2004; Kern & Radford, 2017). Group-living animals can obtain personal information regarding predation risk and feeding opportunities, or utilise social information by monitoring the behaviour and interactions of group members with the environment (Danchin et al., 2004; Dall et al., 2005; Rauber & Manser, 2018). Acquiring social information allows group members to access more information at a faster rate than they would by purely relying on personal information (Giraldeau, 1997; Valone & Templeton, 2002). The use of social information also lowers the energetic and time costs of obtaining information, resulting in fitness benefits (Gil et al., 2017; Giraldeau, Valone & Templeton, 2012). However, individuals within a group may vary in the accuracy and relevance of information they provide (Giraldeau, Valone & Templeton, 2012).

Alarm calls are a form of social information used extensively by group-living species to alert conspecifics to the presence of predators or intruders (Hollén & Radford, 2009; Griesser, 2013). These calls allow individuals to escape imminent danger by either fleeing, or undertaking appropriate defensive measures (Hollén & Radford, 2009; Griesser, 2013). When the costs of ignoring an accurate signal are high (e.g. the risk of predation is high if an alarm call is ignored) inaccurate signalling can be tolerated by receivers without a reduction in responsiveness (Johnstone & Grafen, 1993; Wheeler & Hammerschmidt, 2012). However, when rates of inaccurate signalling are sufficiently high, reacting to the inaccurate signal can be costly (Ydenberg & Dill, 1986; Flower, Gribble & Ridley, 2014). Therefore, it is expected that high rates of inaccurate signalling and high costs of unnecessary response to an inaccurate signal should select for receivers to determine the association between a potential threat and a signal and react accordingly (Maynard Smith & Harper, 2003).

The probability of receivers responding to an alarm call, based on the accuracy of the information provided, has been demonstrated in a number of primate and mammal species including, vervet monkeys (*Cercopithecus aethiops*, Cheney & Seyfarth, 1988), tufted capuchin monkeys (*Cebus apella nigritus*, Wheeler, 2010) and Richardson’s ground squirrels (*Spermophilus richardsonii*, Hare, 1998; Hare & Atkins, 2001). In these studies, individuals were able to associate a caller’s identity with their past calling behaviour. Receivers were therefore able to adjust their behavioural response accordingly, reducing the energetic costs of fleeing and the lost feeding opportunities that result from responding to inaccurate social information (Ydenberg & Dill, 1986; Hare & Atkins, 2001; Blumstein, Verneyre & Daniel, 2004; Beauchamp & Ruxton, 2007).

Although there is considerable evidence of intraspecific differences in the accuracy of signalling behaviour (Munn, 1986; Moller, 1988; Ridley & Raihani, 2007; Flower, Gribble & Ridley, 2014), empirical evidence of the way that receivers respond to such information is relatively rare in avian species (McDonald, 2012; Nichols & Yorzinski, 2016). The Australian magpie (*Cracticus tibicen*) is a group-living songbird that exhibits both cooperative vigilance and territorial defence within established groups (Kaplan, 2004). Vocal signals given by individuals alert group members to threats and invoke territory defence behaviours (Kaplan, 2004; Kaplan et al., 2009; Walsh et al., 2019). The Western Australian magpie is a subspecies of the Australian magpie, and forms social groups that cooperate in both predator and territorial defence (Mirville, Kelley & Ridley, 2016; Ashton, 2017). This subspecies is only found in the south-west of Western Australia and is largely a terrestrial forager (Ashton et al., 2018). Group size ranges between three and 15 adults that defend a territory year round (Johnstone & Storr, 2004; Mirville, Kelley & Ridley, 2016).

Magpies have an extensive vocal repertoire composed of a variety of different call types that vary in function and structure (Kaplan, 2004; Kaplan et al., 2009; Walsh et al., 2019). These include carols (commonly used in a group chorus), warbles and alarm calls (Kaplan, 2004; Farabaugh & Brown, 1991). Magpies are a suitable avian species to study discrimination between alarm signallers because magpies have individually distinct calls that encode information about both individual identity and sex (Farabaugh & Brown, 1991).

To determine whether a reliable association between a predator threat and an alarm call affects vigilance behaviour in response to a signal, we conducted playback experiments (hereafter association experiments) where individuals were exposed to alarm calls from two individuals from their social group. The calls of one individual were played back in the presence of a model predator, while the calls of the other individual were played back in the absence of a model predator. Vigilance behaviours were recorded both before the playback commenced, during the playback (first, fourth and final call) and after the playback had ceased. This allowed us to examine patterns in vigilance according to (a) the presence and absence of a predator stimuli and (b) behaviour pre- and post-playback of an alarm call in both conditions described in (a) above.

A recall experiment was conducted shortly afterward, where both alarm calls were played back in the *absence* of the predator model. This allowed us to test whether magpies responded differently to alarm calls (given by the same individuals used in the association playback experiment described above) according to the accuracy of the recent alarm calls they were exposed to. We predicted that individuals would show greater levels of vigilance to the playback of individuals whose call was associated with the presence of a model predator in the previous association experiment.

## Materials and Methods

### Study species and sites

We conducted playback experiments on eight free-living magpie groups, ranging in size from four to 15 adults. Average group size in our study population at the time of this study was seven adults (see Table 1 for details of the composition of study groups). Groups used for this study were geographically distinct from each other and located in Guildford, Western Australia (31.9000°S, 115.9730°E) and the University of Western Australia Campus (31.9812°S, 115.8199°E).

**Table 1:** Study group demographics.

Group number	No. of individuals	No. of males	No. of females	No. of juveniles	Site
1	12	3	6	3	GUI
2	10	4	4	2	CRW
3	14	6	5	3	GUI
4	10	3	6	1	GUI
5	6	3	2	1	CRW
6	7	5	1	1	GUI
7	7	3	3	1	GUI
8	7	2	2	3	GUI

**Note:**

Site location refers to Crawley (CRW) or Guildford (GUI).

The study population was established in 2013 by ARR. Groups have been habituated to the presence of observers for the last 5 years, allowing observational data to be collected within five m of the birds without affecting their normal behavioural patterns (Mirville, Kelley & Ridley, 2016). Individuals were individually recognisable via a combination of coloured and metal rings (Mirville, Kelley & Ridley, 2016). Western Australian magpies are sexually dichromatic and adults could therefore be sexed based on their plumage patterns and colouration (Mirville, Kelley & Ridley, 2016). Juveniles (<3 years of age) were unable to be sexed based on their plumage patterns and were therefore excluded from this study. Our research protocols were approved by the University of Western Australia Animal Ethics Committee (approval number RA/3/100/1272).

### Call collection

Alarm calls were collected from free-living adults in the habituated study population. Calls were collected from two adult individuals (>3 years of age) per group, who were the same sex, social rank and as similar in age as possible. This allowed us to control for potential biases in receiver response due to the sex or social rank of the caller (Hare, 1998; Kern, Sumner & Radford, 2016; Rauber & Manser, 2018). Calls were collected from a total of 16 individuals across eight groups: four males and 12 females. Out of the calls collected, only high quality calls (those with a high signal: background ratio) were selected for playback. Any calls where other group members were audible in the background were excluded.

Calls were recorded from June to July 2017, between 7 and 11 am, the time period when individuals are most actively foraging (Edwards, Mitchell & Ridley, 2015). The focal individual was followed and a call recorded each time it was emitted. The context of the call (location of bird, type of predator, or identified threat present) was recorded by the observer, noting time, date and location of the caller. All recorded calls were naturally emitted by focal individuals (i.e. no stimuli were used to elicit the calls). Calls were recorded using a RØDE NTG-2 directional microphone set within a Blimp suspension windshield system which was attached to a Roland R-05 wave/MP3 recorder, set at a sampling rate of 44.1 kHz. When collecting calls, the microphone was pointed directly at the bird at a standardised distance of approximately 10 m.

### Stimuli preparation

Playback sequences were prepared using the programme Audacity^®^ 2.1.3. Each playback sequence comprised the alarm call of a group member, a 30 s break and then the repetition of the alarm call from that same group member every 30 s. The alarm call was repeated eight times per playback sequence. Since magpie alarm calls can vary according to predator type (i.e. terrestrial or aerial), and urgency (proximity of predator), we repeated the same alarm call type throughout the playback, to prevent presenting conflicting calls within the playback (Kaplan et al., 2009; Koboroff, Kaplan & Rogers, 2013). Only alarm calls directed at terrestrial predators were used for playback. During the break between calls, background noise (that had been recorded whilst collecting calls) was used. Average alarm call duration was 1.85 s (range 1.66–1.96 s). Since alarm calls can vary in urgency (distance to predator), type (aerial or terrestrial) and location of the caller (ground or elevated), it proved very difficult for us to get multiple high quality exemplars of the same alarm call type under the same conditions for all focal individuals. Therefore, following the protocol of Engesser et al. (2018), to avoid presenting variable and contextually inconsistent alarm calls, we played back one exemplar per individual per playback.

Playbacks were saved as WAV files and transferred to a mobile phone attached to a Braven BRV-1M speaker. Before the commencement of playback experiments, the playback volume was measured so that it corresponded with the natural volume of magpie calls given at 10 m. A DIGITECH Micro Sound Level Meter was used to measure the volume of magpie calls from a distance of 10 m, revealing peak volumes between 60 and 70 db. The playback sequences were then measured using the sound level metre to ensure that volumes also ranged from 60 to 70 dB 10 m away from the speaker. All playback testing was conducted in a location distant from the groups to prevent birds hearing the alarm calls during this measuring period. Each playback was normalised to −10 dB, so playbacks fell within the 60–70 dB range. Normalisation ensured all tracks had the same peak volume and therefore that birds were reacting to call content rather than volume.

The predator model used during the playback sequence was a rubber snake that resembled a dugite (*Pseudonaja affinis*), a relatively common snake species endemic to Western Australia (Judge et al., 2002) and present at our study site. This was considered a suitable predator because magpies are terrestrial foragers, and previous studies have used snakes to elicit anti-predator behaviour in magpies, including mobbing and alarm calling (Koboroff, Kaplan & Rogers, 2013). Thus, snakes pose a conceivable threat to foraging magpies (Koboroff & Kaplan, 2006). We have observed natural encounters between magpies and snakes at our study site. During these encounters, magpies were observed to give what we define as a ‘terrestrial alarm call’. This was the call type we used in our playback experiments.

### Playback experiments

#### Presentation of alarm calls

Of the two individuals per group for which we had previously recorded terrestrial alarm calls, one was assigned to the *snake present* (alarm calls played back in the presence of a snake) and the other the *snake absent* (alarm calls played back in the absence of a snake) playback treatment. A third individual from the same group was randomly selected as the receiver of *both* playback sequences. The order of the *snake absent* and *snake present* playback sequence was assigned randomly to each receiver, and there was at least a 20-min gap between the presentations of playbacks.

The receiver was always an adult, but they were a different sex to the individuals in the playback in five groups due to limited numbers of group members (therefore in some groups there were not three adult birds of the same sex available). A total of eight focal birds were chosen, one from each group. Two of these were female and the remainder were male. Each focal bird received playbacks only from existing group members, in no cases were extra-group or dispersed individuals played back to a focal bird.

Playback presentations were conducted from August to September, 2017, from 7 to 11 am. Playbacks did not commence until focal individuals displayed natural foraging behaviour for at least 1 min (i.e. were not showing signs of vigilance or agitated movement due to external stimuli). Playbacks were presented at a distance of 10 m away from the bird from a Braven BRV-1M speaker that was on the ground and oriented towards the focal individual. The playback individual was always observable so that the direction of the call was coming from where the focal individual expected that caller to be. The birds are habituated to our presence and therefore pay little attention to us. We did not stand directly in front of the playback individual, but near to it, so that the call was coming from the expected direction. We always checked that the playback bird was visible to the focal bird for each playback.

For the presentation of the *snake present* alarm calls, the snake was hidden from the view of the magpies during set up (*sensu* Hare & Atkins, 2001) and not exposed until the alarm call was presented. Fishing line was attached to the head of the snake and the snake was gently pulled at each alarm call to replicate the movement of a snake within grass (Koboroff & Kaplan, 2006). The snake was presented for all eight alarm calls. At the end of the playback the snake was once again hidden. The snake was placed within five m of the focal individual so it was continuously visible throughout the playback. The snake was present in the same location but not exposed during the presentation of the *snake absent* alarm calls.

The behavioural response of the focal individual to the playback sequences was manually recorded with a Samsung Galaxy Tab A tablet using the Prim8 programme, where every behaviour type the bird exhibited during the playback was logged in seconds. Behaviours recorded immediately before, during and after playback were; duration of vigilance, vocalisations, foraging, movement and flying events (*sensu* Koboroff & Kaplan, 2006; Engesser, Ridley & Townsend, 2016). Birds were considered vigilant when they demonstrated an erect posture and were actively scanning for predators (*sensu* Ridley, Nelson-Flower & Thompson, 2013). Resting behaviour such as preening, beak wiping and perching were also recorded. The behaviour of the bird was recorded 1 min before the commencement of playback, over the duration of the playback (4 min) and 1 min after the completion of the playback. This allowed us to establish pre-playback behaviour, their response during the playback, and post-playback behaviour.

#### Do magpies respond to prior alarm call information?

To determine whether the prior association between an alarm call and a predator stimulus affected the subsequent response of receivers, we played back the *snake present* and *snake absent* alarm calls to the same focal individual 10 min after the completion of both playback sequences. The recall playback consisted of a single alarm call given by the *snake present* and *snake absent* caller (caller identity was the same for both treatments as in the first experiment described above), followed by a 1-min break, then the other alarm call was played back (the 1-min break is between the end of the post-playback behavioural data collection for the first playback, and the initiation of the second playback). The order in which the alarm calls were played was randomly assigned to each receiver, and both calls were played back without the presence of the predator (*sensu* Blumstein, Verneyre & Daniel, 2004). Using the same protocol as the first playback experiment, the focal bird was exposed to the playback while in a clearly visible area, and the playback was presented at a distance of 10 m away from the bird while it displayed natural foraging behaviour. The behaviour of the focal individual was recorded 1 min before, during and 1 min after the playbacks using Prim8.

### Statistical analysis

All statistical analyses were conducted in SPSS version 25 (IBM Corporation, 2017) and differences were considered significant when *P* < 0.05.

The proportion of observation time spent vigilant by the focal birds were compared after the initial alarm call for each playback treatment to make sure that the calls from the two different signallers did not elicit different responses at the start of the playback sequence. To determine if there was a difference in vigilance behaviour between focal birds hearing playbacks of alarm calls with *snakes present* and *absent* we compared the proportion of time focal individuals spent vigilant 30 s after the first, fourth and final alarm calls between the *snake present* and *snake absent* playback sequence. We used a sequence of eight alarm calls to replicate the alarm call sequences we typically observe in the field, and to look at how responsiveness changes over time according to the association of an alarm call with a detectable predator threat. To assess differences in behaviour before and after each playback sequence, we compared time spent vigilant 60 s before the start of the playback sequence and 60 s after the completion of the playback sequence for each playback type.

We investigated the terms influencing the proportion of observation time that magpies invested in vigilance behaviour using linear mixed models (LMMs), with treatment (*snake present* or *snake absent*), sequence (the order of the call within the playback sequence—first, fourth and eighth alarm call), and the interaction between these two terms, as predictor terms. We also included the order of playback types to each focal individual (*snake absent* or *snake present*) as a predictor term to test for exposure effects (i.e. to determine whether an individual that received the *snake present* treatment first is more likely to be vigilant during the second (*snake absent*) treatment). Individual and group identities were included as random effects. Analysis was conducted on 48 post-playback behavioural observations from eight birds in eight groups (the sample size of 48 comes from the vigilance behaviour of eight birds measured at three stages (first, fourth and eighth alarm call in the sequence (eight focal birds × three measurement points) for two treatment types (*snake present* and *snake absent*). Proportional data were arcsine-square root transformed to achieve normality.

To determine whether time invested in vigilance behaviour after playback changed compared to vigilance behaviour prior to playback, we conducted an LMM analysis of the proportion of time spent vigilant with treatment (*snake present* or *snake absent* playback) and time (before or after playback), and the interaction between these two parameters, as predictor terms. Individual and group identities were included as random effects. Analysis was conducted on 32 behavioural observations (comprising 16 *before* playback sequence and 16 *post-*playback sequence observations) on eight birds (two playback sequences per focal bird) from eight groups.

Model selection using the Akaike’s information criterion corrected for small sample size (AICc) was employed to determine the model/s that best explained the patterns of variation in the data. Using AICc with maximum likelihood estimation, a series of models were tested, with each model representing a biological hypothesis. Lower AICc values represented more parsimonious models, as per Johnson & Omland (2004). Collinearity between predictor terms was checked using Variance Inflation Factors (VIF). Any variables with a VIF greater than four were considered to be highly collinear, and no collinear terms were included in the same model (Hill & Gelman, 2007; Harrison et al., 2018). Only those terms with confidence intervals that did not intersect zero were considered significant predictors of data patterns (Pan, 2001; Burnham & Anderson, 2002).

Within-individual paired *t*-tests were used to compare the differences in the proportion of time spent vigilant both 30 and 60 s after the recall playback.

## Results

**Figure 1:**
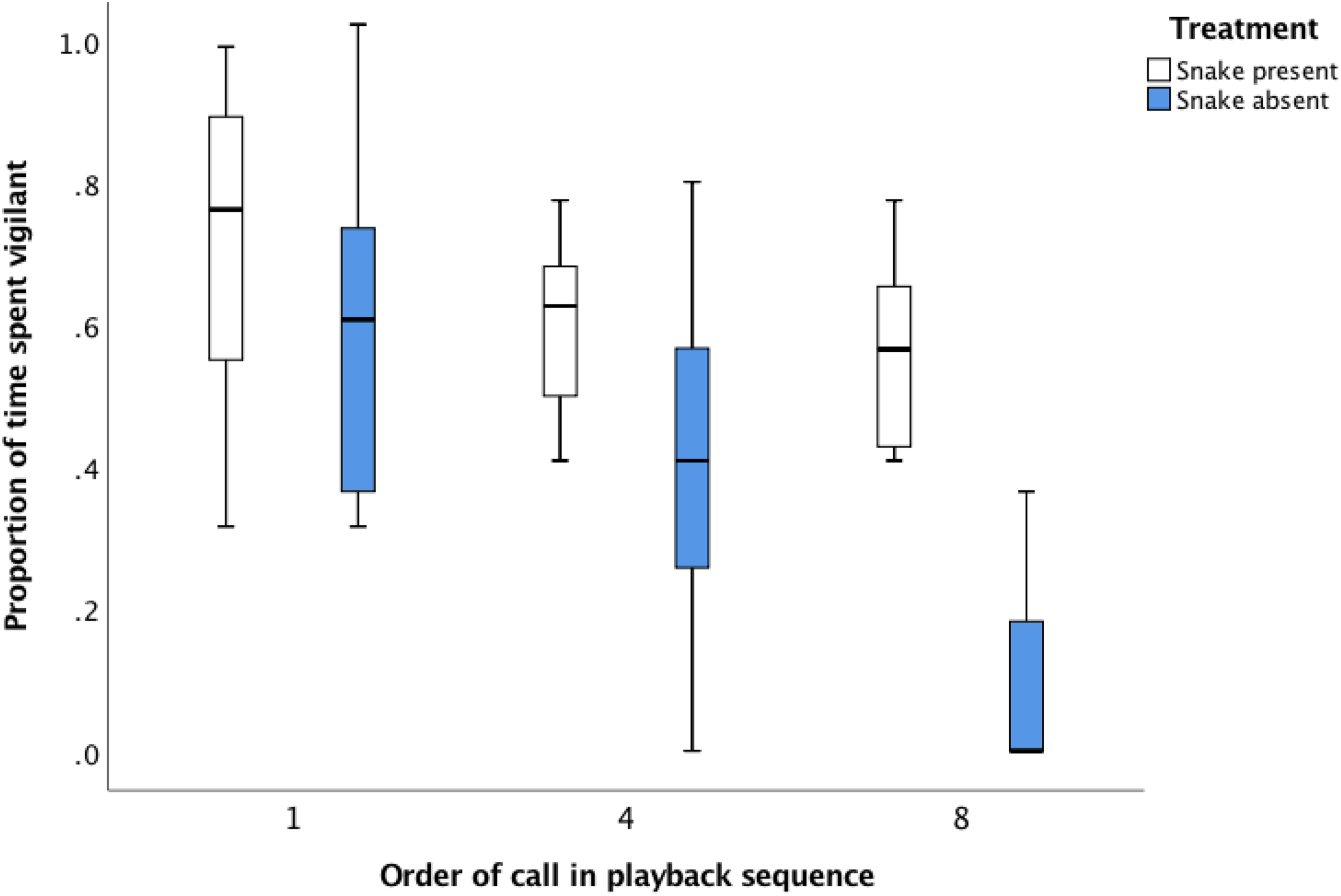
The average proportion of time (median, upper and lower quartile) that focal magpies spent vigilant 30 s after the initial, fourth and final alarm call during the *snake present* and *snake absent* playback sequences.

The proportion of time that individuals spent vigilant declined in response to the *snake absent* playback sequence over the eight alarm calls (Fig. 1; Table 2: Proportion of time spent vigilant after first *snake absent* call: 59.4% ± 6.1%, proportion of time spent vigilant after eighth *snake absent* call: 9.1% ± 12.3%). In contrast, vigilance levels were maintained throughout the *snake present* playback sequence, resulting in no difference in the proportion of time spent vigilant between the first and final alarm call (Fig. 1; Table 2: proportion of time spent vigilant after first *snake present* call: 71.6% ± 6.1%, proportion of time spent vigilant after eighth *snake present* call: 56.1% ± 12.3%). The lack of difference in vigilance after the first alarm call between treatments (proportion of time spent vigilant after the first alarm call was 71.6% for the *snake present* playback c.f. 59.4% for the *snake absent* playback, Fig. 1) suggests there was no *initial* difference in responsiveness of the focal individual to the alarm calls. A difference emerged *after* the sequence of eight alarm calls was played back (56.1% of time spent vigilant after *snake present* sequence completed, compared with 9.1% of time spent vigilant after *snake absent* sequence completed, Table 2).

**Table 2:** Model selection of the terms influencing the proportion of time spent vigilant during the association playback experiments.

Model	AICc	ΔAICc
Treatment	12.47	4.42
Playback order	19.44	11.39
Sequence	11.14	3.09
Treatment + playback order + treatment × playback order	16.96	8.91
**Treatment + sequence + Treatment × Sequence**	**8.05**	**0.00**
Basic	16.49	8.44
**Parameter estimates**	**Estimate**	**95% CI**
Intercept	0.33	[−0.12–0.19]
Treatment × sequence		
With snake		
*Snake present* 1st call	0.62	[0.30–0.95]
*Snake present* 4th call	0.51	[0.19–0.83]
*Snake present* 8th call	0.47	[0.31–0.63]
No snake		
*Snake absent* 1st call	0.50	[0.18–0.83]
*Snake absent* 4th call	0.32	[−0.01–0.64]
*Snake absent* 8th call	0.0	–

**Note:**

Individual identity is included as a random term in the model. Sequence refers to the point in the playback (first, fourth or eighth call) at which vigilance behaviour was measured. The basic model is the model with no predictor terms present. The most parsimonious model is highlighted in bold. *N* = 48 post-playback responses by eight focal birds from eight groups.

**Figure 2:**
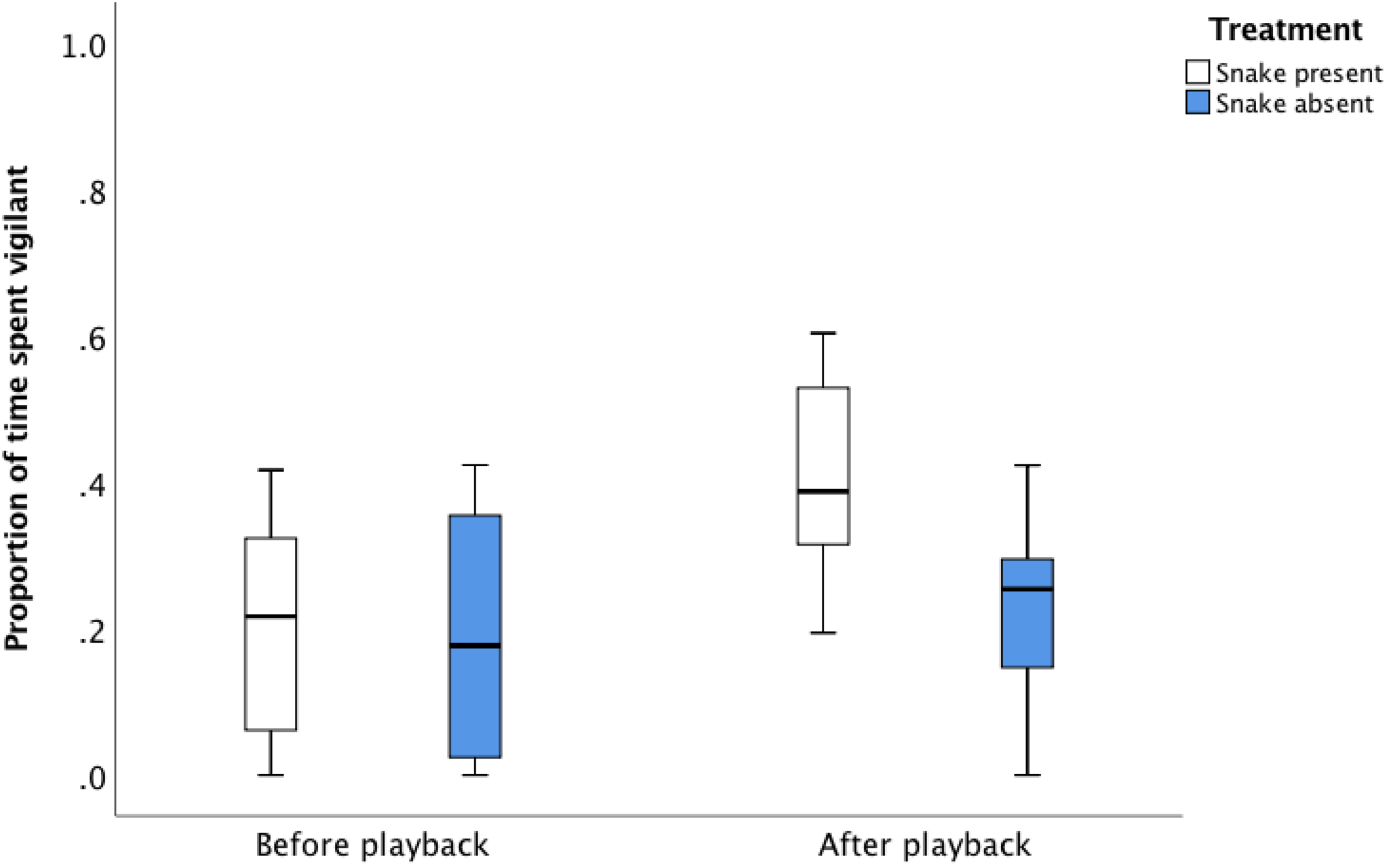
The average proportion of time (median, upper and lower quartile) focal birds spent vigilant 60 s before and after the *snake present* and *snake absent* playback sequence.

There was no difference in the vigilance behaviour of the focal individuals between treatments prior to playback, but there was a difference post-playback (Fig. 2; Table 3). Following the playback sequence of the eight alarm calls in the presence of the snake, individuals increased vigilance in the 1 min after the sequence completed, compared to the 1 min before the sequence started (Fig. 2; Table 3, proportion of time spent vigilant prior to playback: 12.2% ± 1.2%, after playback: 26.8% ± 1.7%).

**Table 3:** Model selection of the terms influencing the proportion of time spent vigilant before and after the recall playback experiment.

Model	AICc	ΔAICc
Basic	248.32	24.82
Treatment	241.27	17.77
Playback order	243.46	19.96
Time	239.86	16.36
**Treatment + time + treatment × time**	**223.50**	**0.00**
Playback order + treatment + playback order × treatment	230.03	6.50
**Parameter estimates**	**Estimate**	**95% CI**
Treatment × time		
*Snake present* before playback	0.63	[−9.30–10.55]
*Snake present* after playback	15.28	[5.35–25.20]
*Snake absent* before playback	0	–
*Snake absent* after playback	2.10	[−7.83–12.02]

**Note:**

Individual identity is included as a random term in the model. The basic model is the model with no predictor terms present. The most parsimonious model is highlighted in bold. *N* = 32 vigilance observations on eight focal birds from eight groups for two experimental treatments (*snake present* and *snake absent*), with two observation periods per treatment (before playback and after playback).

In contrast, following the playback sequence of eight alarm calls in the absence of the snake, there was no change in vigilance in the 1 min after the sequence was completed compared to the 1 min before the sequence started, suggesting no perception of increased predator risk following the completion of the *snake absent* treatment (Fig. 2; Table 3, proportion of time spent vigilant prior to playback: 11.5% ± 1.3%, after playback sequence: 13.6% ± 0.9%).

**Figure 3:**
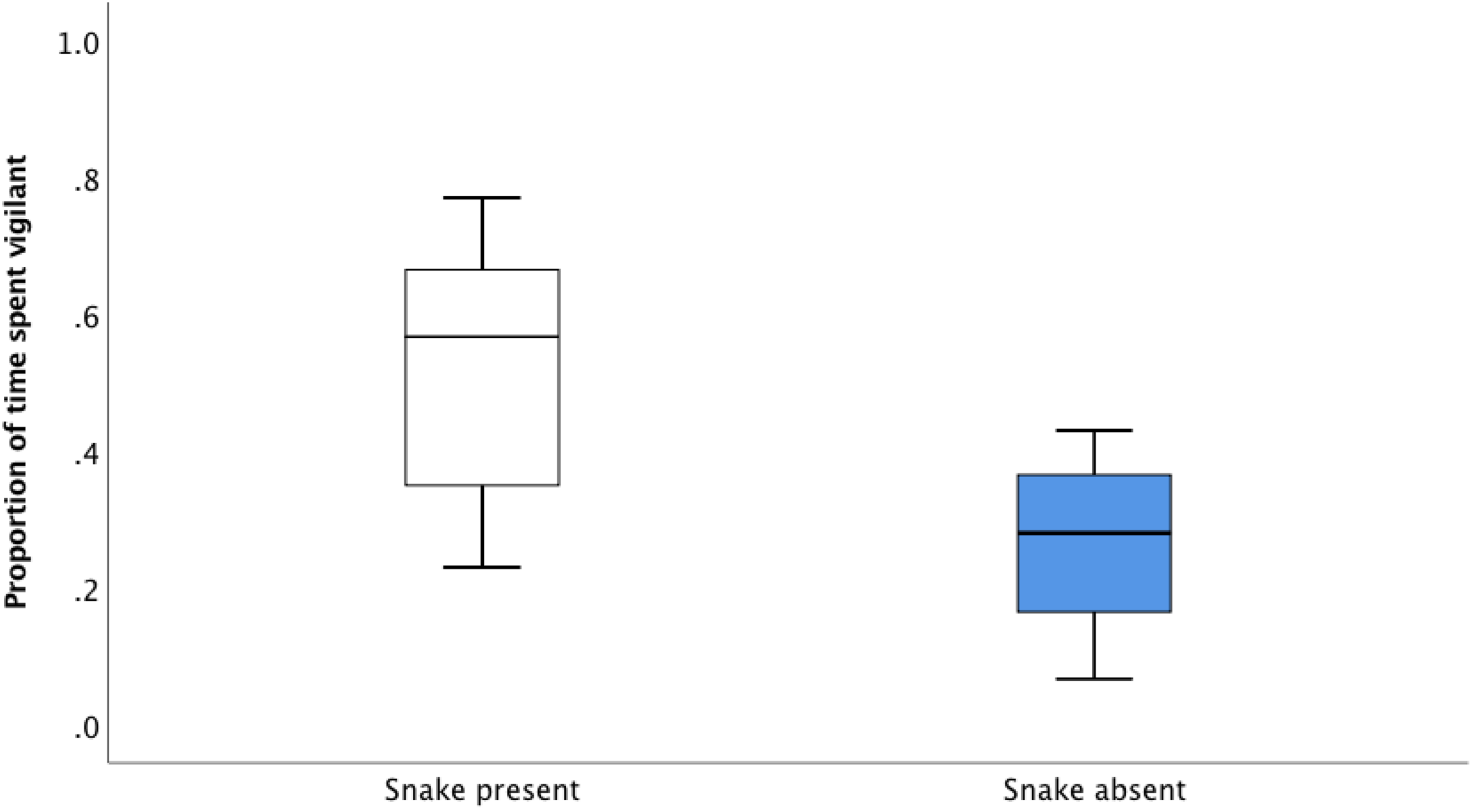
The average proportion of time (median, upper and lower quartile) spent vigilant 30 s after the *snake absent* and *snake present* alarm call during the recall playbacks.

In the recall playback, prior alarm call accuracy (established in the association playback experiments described above) affected subsequent responsiveness to alarm calls. Magpies spent a significantly greater proportion of time vigilant 30 s after the *snake present* alarm call compared to the *snake absent* alarm call (Fig. 3; Paired *t*-test: *t*_7_ = 3.329, *P* = 0.013). This responsiveness was maintained 60 s after the playback (Paired *t*-test: *t*_7_ = 2.421, *P* = 0.046). Although focal individuals showed lowered levels of responsiveness to the *snake absent* alarm call, it was never completely ignored (Fig. 3).

## Discussion

A significant interaction between treatment and sequence in association playbacks reveals that magpies are able to discriminate between alarm calls associated with predator stimuli. By the eighth alarm call in the playback sequence, receivers invested less time in vigilance when the alarm call was not associated with a predator stimulus. These results suggest that individuals modify their behavioural response to an alarm call in accordance with the level of threat associated with the alarm call (Hare & Atkins, 2001). The lack of a difference in response by the focal individual after the first alarm call in the playback suggests that neither alarm call elicited a stronger response in the focal individual prior the commencement of the playback, but rather differences in response by the focal individual developed during playback due to the predator stimulus associated with the alarm call. Receivers ultimately demonstrated diminishing vigilance to alarm calls given in the absence of the snake. These results suggest that receivers are able to detect and react to differences in the accuracy of threat-based information associated with alarm calls and thus contribute to a growing body of evidence of call discrimination based on the type of information associated with the call (Hare & Atkins, 2001).

The results of our recall playback suggest that discrimination between alarm calls based on prior association with a predator threat persists in the short-term. Vigilance in the recall playback was significantly greater in response to the *snake present* alarm call compared to the *snake absent* alarm call, indicating that individuals were able to recall the recent information associated with different alarm calls and adjust their behavioural response accordingly. This could allow receivers to optimise the trade-off between foraging and vigilance behaviour, and potentially minimise the energy expended in responding to inaccurate or irrelevant information (Ydenberg & Dill, 1986; Maynard Smith & Harper, 2003; Scott-Phillips et al., 2012).

Individually distinct alarm calls may allow receivers to detect reliable and unreliable signallers (Cheney & Seyfarth, 1988; Hare & Atkins, 2001; Kern, Laker & Radford, 2017; Rauber & Manser, 2018). For example, over the duration of a playback series, Richardson’s ground squirrels progressively spent less time vigilant after hearing alarm calls from unreliable individuals, as opposed to reliable calls where vigilance was maintained (Hare & Atkins, 2001). Similarly, in vervet monkeys, receivers were able to assess signaller reliability based on their reliability history, and calls from unreliable individuals elicited lower responsiveness than reliable calls (Cheney & Seyfarth, 1988). Thus, recognising unreliable alarm signallers may minimise the energy costs in responding to such individuals, providing more time and energy for other vital activities (Ydenberg & Dill, 1986; Beauchamp & Ruxton, 2007). Although our results found that magpies were able to discriminate between alarm calls associated with predator stimuli, further research is needed to determine whether they discriminate between individual alarm callers.

The fact that receivers did not completely ignore *snake absent* alarm calls supports the theory that ignoring alarm calls and solely relying on social information could be costly (Johnstone & Grafen, 1993; Maynard Smith & Harper, 2003; Kern & Radford, 2017). A signalling system requires a certain degree of reliability to remain evolutionarily stable, and receivers are expected to ignore a signal that it is no longer beneficial to respond to (Maynard Smith & Harper, 2003). However, where there is a high cost of not responding to a signal (e.g. predation), receiver responsiveness can be maintained, even when unreliable signalling occurs (Hauser, 1996). This may explain why magpies still maintained some vigilance after the *snake absent* playback.

## Conclusion

Our research revealed that magpies were able to assess the accuracy of social information, and shift their behavioural response according to the accuracy of such information, similar to behaviour observed in meerkats (Rauber & Manser, 2018), babblers (Ridley & Raihani, 2007; Flower, Gribble & Ridley, 2014) and weavers (Baigrie, Thompson & Flower, 2014). These results support the mounting evidence that individuals are able to discriminate between signals based on the type/quality of information that each signal provides (McDonald, 2012; Nichols & Yorzinski, 2016; Colombelli-Négrel & Evans, 2017).

## Supplemental Information

10.7717/peerj.7572/supp-1 Supplemental Information 1 Data for playback response. Behavioural responses to playback per individual

## Additional Information and Declarations

### Data Availability

The following information was supplied regarding data availability: The raw data is available as a Supplemental File.
